# Masculinity and mental health treatment initiation for former political prisoners in Yangon, Myanmar – a qualitative investigation

**DOI:** 10.1186/s12889-021-10249-2

**Published:** 2021-01-25

**Authors:** Daniel P. Lakin, Kyaw Soe Win, Htin Aung, Khin Nyein Chan Soe, Bo Kyi, Arik V. Marcell, Wietse A. Tol, Judith K. Bass

**Affiliations:** 1 Department of Mental Health, Johns Hopkins Bloomberg School of Public Health, 624 N Broadway, Suite 780, Baltimore, MD 21205 USA; 2 Assistance Association for Political Prisoners, No. 75 Oakthaphayar Street, 33 Extension Ward, North Dagon Township, Yangon, Myanmar; 3 Johns Hopkins University School of Medicine, 200 N Wolfe Street, Baltimore, MD 21287 USA

**Keywords:** Political prisoners, Mental health, Men, Mental health services, Myanmar, Burma

## Abstract

**Background:**

Men living in low- and middle-income countries are unlikely to seek mental health care, where poor healthcare infrastructure, differences in illness conceptualization, and stigma can impact treatment seeking. Vulnerable groups, such as former political prisoners, are more likely than others to experience potentially traumatic events that may lead to negative mental health outcomes. To improve the likelihood of successful engagement of vulnerable men in psychotherapy, it is necessary to identify factors that influence treatment adherence, and to better understand men’s attitudes surrounding decisions to seek and initiate care. The purpose of this investigation was to explore themes of masculinity, treatment seeking, and differences between male former political prisoners who accepted and declined therapy in an urban low-income context.

**Methods:**

We conducted a qualitative, interview-based investigation with 30 former political prisoners in Yangon, Myanmar who were eligible to receive mental health counseling provided by the non-governmental organization (NGO), Assistance Association for Political Prisoners. Men were initially screened using a composite questionnaire with items related to depression, anxiety, and posttraumatic stress symptom severity. After screening, if potential clients were identified as having probable mental health problems, they were asked if they would like to participate in a multi-session cognitive behavioral therapy program. Semi-structured, open-ended interviews were conducted with 15 participants who accepted and 15 participants who declined therapy. Interviews were transcribed and translated by local partners and thematically coded by the authors. We used thematic analysis to identify and explore differences in treatment-seeking attitudes between men who accepted and men who declined the intervention.

**Results:**

Men described that being a community leader, self-reliance, morality, and honesty were defining characteristics of masculinity. A focus on self-correction often led to declining psychotherapy. A general lack of familiarity with psychological therapy and how it differed from locally available treatments (e.g. astrologists) was connected to stigma regarding mental health treatment.

**Conclusions:**

Masculinity was described in similar terms by both groups of participants. The interpretation of masculine qualities within the context of help-seeking (e.g. self-reliance as refusing help from others versus listening to others and applying that guidance) was a driving factor behind men’s decision to enter psychotherapy.

## Background

An estimated 75% of people in need of mental health services in low- and middle-income countries (LMICs) do not receive treatment [1]. While the burden of mental health problems is borne largely by LMICs [1, 2], treatment infrastructure for such problems is largely underdeveloped in these contexts [3, 4]. To address this treatment gap, research and practice have focused on culturally adapting evidence-based interventions and training lay workforces to provide mental health services in non-specialist settings [5, 6].

Despite a similar prevalence of general mental health problems for men and women in both low- and high-income countries [7], men are less likely than women to seek formal treatment for mood and anxiety disorders [8]. Studies conducted in high-income settings indicate that men are less likely to hold favorable attitudes towards care [9], and are more likely to drop out of interventions for post-traumatic stress (PTS) [10] and depression [11, 12]. In LMICs, greater exposure to stressors, such as violence and poverty, can exacerbate the likelihood of poor mental health for vulnerable groups.

Men in low-income and humanitarian contexts face considerable barriers to engagement with mental health services. A systematic review and meta-analysis of mental health-related stigma and help-seeking found that the interplay of societal norms and attitudes towards care were a driving force behind limited help-seeking behaviors for men [13]. While these attitudinal barriers to men’s engagement with mental health services have been identified in high-income contexts, the extent to which these factors are important in LMICs has received little attention. In these settings, psychological distress among men has been associated with an increased likelihood of living in poverty, abusing alcohol, and perpetrating gender-based violence [14, 15]. The little research that has focused on men affected by violence has largely focused on former combatants [16], and not on other men in the community in need of services. In a recent systematic review of 29 trials of mental health treatments in LMICs, four focused specifically on men – largely ex-combatants – and five included 50% or more men in the sample [16]. Extant literature from high- and low-income contexts [9, 17–20], including multiple trials [21–24], has reported engaging and retaining men in psychotherapy. Some reported significant treatment effects only for women [23, 25]. Cultural adaptations of evidence-based treatments [26, 27] and diagnostic practices [28] have been shown to successfully improve mental health in various socio-cultural settings [6, 29], however, male gender identity does not appear to have been considered in such adaptations [6].

There is an established link between political imprisonment and the development of PTS and other severe mental health sequelae [30–32]. Few studies to date have specifically investigated the mental health needs of former political prisoners in Burma [33, 34], but extant literature notes the importance of cultural considerations that emphasize local conceptualizations of mental health problems and treatment. Political prisoners in Burma are subjected to torture, inhumane conditions, forced labor, and violence throughout their incarceration. Upon release they face adversity due to joblessness, poor economic conditions, and a permanent criminal record for political dissent present. In Yangon, the largest city and site of this study, formal mental health treatment is handled primarily by Yangon Hospital, and typically relies on poorly monitored psychotropic medication or outdated and harmful inpatient practices, e.g. isolation or confinement [35]. Mental health and psychosocial support (MHPSS) programs, and counseling in general, are often considered novel and do not have widespread support or recognition. Community-level measures are typically limited to familial/social support and local astrologists who rely on the traditional Burmese zodiac to provide solutions to common problems and advice for stress management. In recent years, community-based organizations (CBOs) have collaborated with non-governmental organizations (NGOs) and universities to disseminate, implement, and evaluate mental health programs for traumatized populations in both urban Yangon and rural areas [35].

We sought to gather information from the perspectives of a group of former political prisoners regarding the decision to accept or decline mental health treatment. We were interested in examining political prisoners’ views on masculinity, and to exploring treatment seeking and attitudinal differences between male former political prisoners who accepted and declined psychotherapy in an urban setting within a low-income country – Yangon, Myanmar. Formative investigations that identify mutable individual characteristics of the population of interest, as well as features of the treatments themselves, are critical to the development of treatment protocols that are better suited for engaging and retaining men.

### Background and setting

Burma or Myanmar is a union of seven states corresponding to minority-held territories, and seven divisions populated mostly by the largest ethnic group, the Bamar. It has a population of 51 million people, with roughly 70% living and working in rural areas, and 30% living below the national poverty line [36]. Theravada Buddhism, the state religion since 1961, is prominent across Burmese cultures [37]. Two central components of Theravada Buddhist doctrine include the Five Precepts and the Eightfold Path. The Five Precepts, are five moral doctrines involving abstinence from: harming living things, sexual promiscuity, stealing, lying, and substance abuse. The Eightfold Path, one of the Four Noble Truths, encompasses the core doctrine of Buddhist practice for escaping suffering and the cycle of rebirth. It consists of eight guidelines (e.g. Right Thought or Right Speech) that serve as a means by which followers can cultivate moral and intellectual virtue through mindful living, and ultimately, escape from the cycle [38].

Burma has faced decades of political and economic instability since its independence after World War II. In 1987, Prime Minister Ne Win mandated the abandonment of several denominations of currency rendering nearly 80% of Burmese currency valueless [39]. The timing of the devaluation was such that Burmese university students could not afford upcoming tuition payments, leading to student revolts, university shutdowns, and violent confrontation with police. Casualty estimates from some non-governmental organizations (NGOs) were higher than one thousand civilians killed in Yangon alone over only 3 days during the height of the protests [39]. Students who participated in the dissemination or production of pro-democracy materials – or who were rumored to – were charged with sedition and incarcerated. This crackdown spread across Burma, leading students to join ethnic militias in minority-held states to combat the central government. Others, fearing continued violence and political persecution, fled to neighboring Thailand and other countries. Many who stayed in-country were jailed as political prisoners.

While the national constitution was redrafted in 2008 and transitioned to partial civilian rule, the Burmese government still does not acknowledge that it has or has ever had political prisoners. Following additional protests in the 1990s and 2000s, a second generation of political prisoners was incarcerated as the Burmese central government continued to arrest university students for participating in pro-democratic rallies. Since the National League for Democracy won major elections in 2012, 35 political prisoners have been convicted, 56 are in jail awaiting trial, and 235 are awaiting trial on bail [40].

## Methods

The interviews for this study, conducted between July and September 2018, took place within Yangon proper and in nearby Thanlyin township at Thabarwa Meditation Center and Hospital - a *yeikthar,* or monastery and education center - providing basic medical and housing services for homeless clients. Interviews were conducted by three interviewers (one female, two male) employed by the Assistance Association for Political Prisoners (AAPP), a local NGO, as part of standard client recruitment for AAPP’s mental health assistance program (MHAP). All interviewers were former political prisoners themselves. Interviewers were trainers of trainers (TOTs) in the Common Elements Treatment Approach (CETA) who received their initial training as part of a continuing partnership with Johns Hopkins University [33, 41]. CETA is a 12-sessioncognitive-behavioral therapy designed to address depression and PTS among participants [42]. Transcription of the recorded interviews was provided by three AAPP CETA counselors fluent in Burmese. The interviewers and transcribers attended a two-day, qualitative research methods training at the AAPP office. The training covered basic principles of in-depth interviews (e.g. probing questions), as well as an introduction to the study aims and interviewing materials.

Ethical approval was provided by the Johns Hopkins University Institutional Review Board prior to data collection. A local advisory board comprised of mental health professionals from an AAPP partner clinic serving Burmese refugees and former political prisoners living in Mae Sot, Thailand also approved of the interview and recruitment materials in June 2018.

### Interview procedures

The in-depth interviews themselves were semi-structured and lasted between 30 and 60 min. To discuss participant recruitment strategies, interview themes, and interview fidelity, the primary author facilitated weekly group supervision meetings with all three AAPP counselor/interviewers. In these sessions, the interviewers described one or two of the interviews they had conducted in the past week, the content of those interviews, what questions from the interview were well-received and which did not yield rich responses from their clients. These conversations shaped subsequent interviews, as questions were added or removed from the semi-structured prompts depending on interviewer feedback. After transcription by AAPP staff, the subsequent anonymized transcriptions were translated into English by two non-AAPP professionals in Yangon.

### Participants

Interviews were conducted with 30 adult men (age ≥ 18 years). Participant ages ranged from 24 years to 72 years. All men interviewed were former political prisoners who spent between six and 25 years incarcerated for sedition against the Burmese government. A majority of participants were incarcerated in the late 1980’s, while several younger men had been apprehended for speaking out against the military regime in the early 2000’s.

All participants were referred by community members to AAPP counselors as part of their MHAP program. Given that Burma does not acknowledge the existence of its own political prisoners, AAPP maintains a fragile relationship with the government. As such, AAPP worked entirely in Mae Sot with political prisoners seeking asylum in Thailand for 15 years. In 2015, they were removed from the Burmese CBO blacklist and now operate in Yangon. Though they are a relatively new organization in Yangon, they are actively trying to expand their caseloads. They frequently advertise and promote their MHAP program through social media and public events. Many respondents subsequently self-referred based on AAPP’s reputation and word of mouth or were referred by family. Others were friends, neighbors or former inmates imprisoned with the interview staff. Following AAPP’s standard intake process, participants were asked if they would be interested in participating in a second interview to discuss their decision to participate or not participate in CETA. No formal sampling framework was used, as the interviews immediately followed screening for MHAP inclusion criteria.

While most clients were recruited from residential neighborhoods in Yangon proper, several (*n* = 8) were referred by medical staff from the Natural Meditation Center (*Thabarwa Yeikthar*). The *yeikthar* serves as an urgent care center, hospice, and homeless shelter for men and women unable to afford housing or medical care. Respondents from this center were often suffering from highly stigmatized chronic diseases (e.g. HIV) or debilitating conditions (e.g. stroke-induced partial paralysis) resulting in familial abandonment or isolation.

Informed consent to participate in these interviews was obtained immediately following client screening. All potential MHAP clients were screened for potential mental health problems using a questionnaire initially developed for a randomized controlled trial of CETA with Burmese refugees in Mae Sot [33], many of whom were former political prisoners. This locally-validated screener contained 15 items related to depression and 30 related to traumatic exposures and PTS symptoms derived from the Diagnostic and Statistical Manual for Mental Disorders, Fourth Edition [43] criteria for each respective mental health problem. Exposure to specific traumatic events was captured in the PTS section of the screener, but was not discussed in the qualitative interview to mitigate psychological distress. Items were developed and incorporated after extensive community-based participatory research with former political prisoners and refugees to ensure more valid measurement of psychological distress within this context [44].

Respondents reported symptom frequencies for the past month, from 0 (“none of the time”) to 3 (“almost always”). The inclusion criteria for each disorder are listed in Table 1. Participants met criteria for depression if either criterion A or B was met, and 3 or more of the symptoms from criterion C were endorsed with a “2” or “3” rating on the Likert scale. Participants met criteria for PTS if they meet any two of the criteria. Immediately following this assessment, participants were asked if they would like to participate in the MHAP program. To explore potential attitudinal differences between men who accepted and declined therapy, interviews were conducted with both participants who accepted treatment (*n* = 15) and participants who declined (*n* = 15).

**Table 1:** Inclusion Criteria for Depression and PTSD

Depression		PTS	
**Criterion A**	*Response of 2 or 3 on one of these items*	**Criterion A**	*Response of 2 or 3 on one of these items*
Feeling Hopeless		Recurrent thoughts/memories of the event	
Feeling Sad		Feeling as though the event is happening again	
Feeling Lonely		Recurrent Nightmares	
Crying Easily		Sudden emotional/physical reaction when reminded of event	
**Criterion B**	*Response of 2 or 3 on one of these items*	**Criterion B**	*Response of 2 or 3 on one of these items*
Loss of sexual interest/pleasure		Avoiding activities that remind you of the event	
Feeling no interest in things/less interest in daily activities		Inability to remember parts of the most traumatic or hurtful events	
		Avoiding thoughts or feelings associated with the traumatic event	
**Criterion C**	*Total number of the following items endorsed with 2 or 3*	**Criterion C**	*If at least 2 of the following 4 questions have responses with 2 or 3*
Low energy, slowed down OR feeling like everything is an effort		Feeling detached or withdrawn from people	
Poor appetite		Unable to feel emotions OR hard to suppress feelings	
Sleep difficulties		No interest in daily activities	
Thoughts of ending your life		Feeling like you don’t have a future	
Worry to much about things			
Blame self for things OR feel worthless			
		**Criterion D**	*A response of 2 or 3 on one of the following*
		Blame self for things OR feel worthless	
		Feeling jumpy or easily startled	
		Difficulty concentrating	
		Feeling on guard	
		Feeling irritable or having outbursts of anger	

Items were derived from the PTSD Checklist [29], the depression subscale of the Hopkins Symptom checklist [30], and locally-derived items from previous formative work [31]

### Analysis

Data collection and analyses procedures were rooted in two qualitative methodologies – constructivist grounded theory [45] and more general thematic analysis [46]. Grounded theory-based approaches are well-suited to developing a theoretical framework to explore this decisional process (i.e. accepting or declining treatment), and to provide a broader sense of factors that motivate or shape that decision (e.g. masculinity, barriers to care, perceptions of treatment, etc.). Theoretical sampling and in vivo coding could not be adhered to strictly given time, translation, and resource constraints. However, clients and counselors were consulted regularly for insight into improvements in recruitment and data interpretation procedures. Initial coding of some interviews and identifying thematic content was conducted while the first author was in-country when possible, with feedback from the interview staff regarding emergent themes. These themes in turn informed how interview and data collection processes might be modified.

Given the difficulty of translating multiple interviews with precision in a short amount of time, the primary coding for a majority of the interviews was conducted after the first author returned from Burma. For thematic analysis, the transcripts were coded, organized, and analyzed using Nvivo version 12 [47]. The overarching thematic codes, derived with help from the interview staff (authors KSW, HA, KNCS), served as an initial guide for data interpretation. After first reading an interview, the first author coded each transcript line-by-line to explore subthemes and examples, and to further develop the initial broad codes, e.g. conceptualizations of masculinity within the sample. These codes were confirmed or reconciled with help from interviewer KSW and HA. After coding interviews, themes regarding the differences between men who accepted and declined therapy were developed primarily by DL through active collaboration with KSW, WT, & JB.

## Results

Men who accepted and declined therapy described thematically similar norms and expectations with regard to masculinity. As such, the results begin with description of masculinity as defined by the full sample of interviewees, followed by a comparison of themes between groups (i.e. men who accepted and men who declined).

### Masculinity

*Honesty* and *morality* were the most commonly cited core characteristics of masculinity among interviewees (*n* = 21; 70%). Descriptions of morality and honesty were commonly based on the Buddhist Five Precepts. “An honest man” was the title most men strived for among their peers. While honesty was related to speaking truthfully, it was also related to living in a forthright and plain manner, e.g. as described by a 35-year-old participant, someone who is “righteous, [who does things] without discrimination…He must be able to decide fairly, and speak up if [something] is good or bad. He shouldn’t nod for a bad thing if it is good; he needs to justify. He must speak the right thing without cheating. Even his inner mind must be righteous.”

Righteousness and having a “a good moral spirit” were predicated on following the Five Precepts, often verbatim. There were particularly strong feelings against the use and abuse of alcohol and drugs, despite the simultaneous frequent mention of drinking with friends and getting drunk as a coping mechanism for psychological distress. One participant mentioned four of the precepts – abstinence from alcohol, stealing, lying and harming living creatures - in a single response, stating that “to be a gentleman, one must have good morality. For example, he must not lie or steal. He must be humane, and have a spirit of volunteering …In addition, he must not drink…” Only one participant mentioned “not mixing with women,” because they “should do their own business.”

While the Five Precepts are not inherently gendered, men commented on the belief that “[Burmese people] have the concept that men are better and more noble than women,” even though the moral “checklists are not supposed to be ‘this is for men’ or ‘this one is for women.’” However, based on these responses, it seems that Burmese men believe there is an expectation to be models of morality and that they are the ones to uphold these Precepts.

*Breadwinning* emerged as a core feature of Burmese masculinity (*n =* 10; 33%). One interviewee succinctly explained, “A man is someone who needs to provide income for his family…When their wife is making money, I don’t consider that kind of person as a man.” Spending time away from one’s family while incarcerated added an extra dimension of urgency for some participants. As one participant explained:

*As I was away from my family for a very long time [while in prison], I have the feeling that I have the responsibility to take care of my family as much as possible. When I got back [home], I saw that my family’s living was not in good condition, so I had to try so hard to fill that blank.*

While being the primary economic support in a household is a common feature of traditional masculinity [48], there appears to be added pressure to provide economically among these respondents given their status as former political prisoners.

For half the participants (*n* = 15*),* being the primary earner in a household was intrinsically linked to the idea of *leadership* within a community for men – the twofold ability to provide thoughtful, measured solutions to difficult problems, as well as to be recognized as a person of status/importance. Leadership was defined as “doing things bravely in front of other people,” and taking “responsibility and accountability.” Leaders are responsible for addressing prominent issues and advancing an agenda, and not moving “crabwise,” as one participant described it. “There are some in the neighborhood who are neither leading from the front nor following behind, but moving crabwise,” he said. “He should lead from the front if there is something urgent and take care of it right away.” As one participant stated, “just like the Myanmar saying, ‘a big tree is home for a thousand birds.’” This sentiment – that men should serve as pillars in their community and family – is furthered by the belief that leadership is a necessary facet of masculinity among many of the men interviewed.

Indeed, one’s ability to actualize and perform duties related to community needs was directly related to the quality of his character, such that indolent or non-active men were seen as morally deficient. As one 66-year-old participant explained:

*If the person uses [his past] knowledge and experiences for the welfare of his family and his community, he will be a useful person. If the person does not or is not able to use them, that person will be useless and maybe he will have a negative effect on the community.*

This judgement regarding one’s value as measured by his utility is a key feature of the theme of *self-reliance* – men are expected to individually resolve their problems and disputes without outside help. Sixty percent (*n* = 18) of men interviewed described this as a priority. Issues of mental health are no exception, as one participant noted: “When the problem is dealing with mental health, it needs to be fixed by yourself first. After you have fixed it yourself, you can create good works in your environment.” Another respondent summarized the process of internal change as a process of mental fortitude and will. “I have just such a kind of tough mind. I think it’s all related to mentality. Strength of will: when you are not able to walk on your own, you need the will to be able to walk.”

With regard to self-reliance, several respondents noted that men have strong minds, and that there is a tension between the stubbornness borne out of this strength and a pressure for men to appear in control and not to “explode,” or outwardly demonstrate an inappropriate level of anger or sadness. As one man who refused counseling mentioned, “I don’t think they will accept [mental health services]. Some [men] are really stubborn. I try to persuade them to concentrate on other things instead of exploding their anger, but they don’t listen.” According to another respondent, “men suffer more than women [because] they are strong-minded in nature. So they more easily lose their temper, and explode more violently. They might feel sad, and more depressed than women.”

Guilt or shame stemming from accepting others’ help was enough to keep some men from seeking help. There was a common refrain among men interviewed that everyone “has their own problems.” “I’m not the type to depend on others,” one respondent who accepted therapy said, “I want to stand on my own two feet. I feel guilty relying on the support of others.” The same respondent went on to clarify that each man “has the feeling that ‘we must’ whether we want to or not. We also know that we have fewer choices and we must work*.”*

### Accepting and declining therapy

#### Men who declined

Across all 30 interviews, men were asked to describe their reasons for accepting or declining therapy. Men often declined because they felt they were dealing with their problems reasonably well on their own and did not believe psychotherapy would be helpful. Men’s descriptions of plans to manage stressors were often tautological, vague, and terse – “I address…everyday tasks by solving [them]. If I need to write, I write. If I need to discuss, I discuss. If I need to attend a meeting, I attend. That’s all.” Another participant simply said, “When the problem is dealing with mental health, it needs to be fixed by yourself first…If you do good things, you will be good. If you do bad things, you will be bad. That’s it.” The belief that men are expected to solve their problems on their own was summarized by one respondent who declined therapy, who said:

*Men mostly don’t take psychological treatments – they believe too far in the tradition that men are heads of the family, and whatever they do is right. That’s why they don’t easily accept if they have mental or physical problems and moreover, they don’t evaluate themselves, and they are hard to change.*

These quotations suggest that mental health concerns are equated with poor morals in that men who are unable to handle mental problems have probably done something wrong that led to a negative outcome.

Others believed that Burmese people were concerned with not “giv[ing] their problems to others”, as this may increase suffering in their community. “I have my own problems, and everyone has their own problems,” said one respondent, a taxi driver. “Physically, we might struggle to control our anger and fight with others. Mentally, what we have suffered only concerns our own minds.” This sentiment, that what happens in the mind is the burden of the individual underscores the conflict between self-reliance and treatment seeking –to what extent could the involvement of an unfamiliar third party be beneficial? “That’s why I refuse counseling,” one respondent said, “I don’t really want to change myself with the help of others. That’s my belief. I’ll change myself. When you get counseling, you have to follow their advice.” Counseling was seen by several men who declined therapy as an imperious practice that undermines a potential client’s autonomy.

Not having time for therapy or needing to work was also a frequent reason for declining participation. There was a sense that an hour spent in treatment was an hour better spent working - “I can make 15,000 – 20,000 *kyat* during this time…A man has to give priority to making money, so I must refuse counseling.” Another respondent mentioned that he was personally interested in receiving counseling, but “I can’t give time for [it], although you are willing to provide it.” Some were also wary of taking time off work for something that amounted to “30 minutes or an hour to meet up with each other, open up all our sufferings, make small talk, and relieve our feelings. After that we go on our way and work.” Finding even an hour to commit to therapy was decidedly difficult, as unpredictable economic and employment situations render scheduling impossible – “You know my situation, right? Everything is unstable for me.”

Many men mentioned a lack of familiarity with mental health therapy or psychological counseling as another reason for refusing. Men “don’t know what a counselor is, or what counseling services are. They think that counselors are just like anybody else because they don’t wear uniforms like lawyers or soldiers. Counselors are nothing – most of our citizens think like that.” The role of a mental health counselor, or of counseling in general is unclear, even after an introduction to the CETA program.

The role of a counselor was viewed as overlapping with local astrologists – experts in the Burmese zodiac who are often consulted to manage stress from family and work problems. “We have our own superstitions, whether they’re real or not,” one man said. “[People] go to a fortune teller (*nat sayar*) or astrologist. We get help from those kinds of things.” Another participant added that astrologists are “quite good at making someone relieve their stress, and help concentrate on other things.” He added that there is still a need for “scientific and realistic mental treatment in Myanmar.” Astrologists are chiefly consulted by women and girls, according to the respondents. It is conceivable that lack of familiarity with counseling, little understanding of the differences between counseling and astrology, and the assumption that similar community services are primarily for women contributed to men’s abstention from services.

#### Men who accepted

Men who accepted the intervention similarly cited a desire to address their own problems, but with the additional belief that input from a third party would allow for personal development and a greater likelihood of improved well-being. “I have noticed myself and I don’t know what is happening to me,” one participant mentioned. “I’m over 70 years old and I need some support.” Interest in improving well-being and in introspection were the most commonly mentioned reasons for entering treatment among men interviewed. Men’s motivation to engage in psychological therapy was commonly couched in the desire to be self-reliant. Men who accepted therapy believed that it was an opportunity to receive guidance that would enable them to be more self-reliant and useful. One participant stated he “can tell what is on my mind and…the things happening in my surroundings…Counseling is a higher psychology that I can’t reach. It digs down to a deeper question, and if I can get there, I can get much more knowledge. I will be motivated, and be able to raise my life from this experience.” While the desire to appear self-sufficient was cited as a reason not to go into therapy among men who declined, it was also the driving reason behind accepting therapy.

Several participants spoke about their hopes for treatment in terms of symptom reduction (e.g. “I hope to get some advice on how I should think and behave to relieve my feelings”), improving “motivation” (e.g. …I can be psychologically motivated by counseling…), and most commonly a belief that improving one’s mental health would help the individual better serve his community. Men who accepted therapy believed that through being vulnerable, one would “start to think of doing merit for the community.” Like self-reliance, serving others was a reason both to accept and to decline the intervention. While both men who accepted and men who declined therapy stated that everyone in their community was facing the same kinds of problems (e.g. economic, work-related, family stress, etc.), men who accepted the intervention qualified that statement by adding that men “need to be dutiful” to their community, and through therapy men can “benefit [their] community.” The desire for deeper understanding of thoughts and feelings, and curiosity about these internal processes, seem to fundamentally change how existing conceptualizations of masculinity shaped their decision to enter treatment.

Men from the *yeikthar* all suffered from complications from other stigmatizing conditions such as HIV, partial paralysis, or missing limbs. One HIV-positive, *yeikthar-*based interviewee stated that he accepted services from AAPP because they were the first providers to not approach him “wearing masks.” He went on to clarify, “When we go and get our medicine at the clinic, people come wearing masks because they don’t want to be infected.” Given the stigmatized nature of many medical problems, it may be the case that receiving care for one condition led participants to accept care for another condition that is similarly underrecognized by many in Burma.

## Discussion

The men involved in this study were all political prisoners, some of whom were incarcerated for more than 20 years. As part of their screening for MHAP, men described being held in isolation, tortured, and beaten throughout their imprisonment. After their release, they experienced intimidation, stigma, and feelings of persecution by their government. They represent a highly traumatized group, and as such, the extent to which their responses are generalizable to other Burmese men is unknown. Comparing these findings with interviews with non-prisoners could provide a more nuanced description of how much the views of such a highly traumatized population differ from the general public regarding men’s treatment engagement.

All men interviewed were fairly consistent in their depiction of a masculine ideal embodied by honesty and morality, breadwinning, leadership, and self-reliance. Men who accepted and men who declined service both described the importance of self-reliance and community service. However, men who accepted treatment saw the opportunity to work with a third party as a way to become more self-reliant rather than less. Men who declined believed that counseling was akin to being forced to follow someone’s advice, whereas men who accepted believed they would ultimately be gaining new coping skills through professional guidance that would assist them in becoming more self-reliant and serve their communities better.

Masculine identity and the extent to which men endorse specific masculine traits can vary contextually, particularly in treatment seeking contexts [49]. In this study, masculinity as described in both groups seemed capable of helping or hindering the likelihood of entering treatment. That issue has been explored within the context of masculinity in theoretical frameworks that rely largely on data from the developed world (e.g. a prototype/willingness model [50];, and additional research in humanitarian, LMIC, or conflict settings could provide additional detail.

Respondents’ answers provided a prototype for the ‘ideal man’ and could be used to inform how mental health services are subsequently described and defined to men in need. Given a lack of familiarity with mental health problems and MHPSS programming in Burma, service providers may be in a position to develop a robust approach to introducing and recruiting for MHPSS programming that accurately reflects what men expect at the point of initial contact or during outreach. Describing mental health interventions in less prescriptive terms - e.g. avoiding terms that categorize a potential male participant as ill, broken, etc. – and focusing more on how collaborating with a service provider might improve community standing or self-reliance may in turn improve treatment engagement among men.

The current study raises questions related to the potential benefits of incorporating men’s conceptualizations of masculinity into MHPSS programming, and how that might inform working descriptions of a given intervention. An example might be framing CETA as an opportunity to help participants become better providers for their family through problem solving, help them be seen as more reliable fixtures of their community, or use cognitive skills to solve their own problems in a meaningful way. Future research might include community-based participatory research with men that explores both conceptualization of masculinity and acceptability of mental health treatments. Program materials for existing or novel interventions (e.g. recruitment flyers, consent forms, or therapy principles) could be developed to match the specific language used by men in the community when describing goals for wellbeing, and assessed for differences in acceptance and attrition among men.

### Limitations

This study was limited in that the interviewers responsible for primary data collection were all trained mental health professionals. This is a potential source of bias, as they are all actively supportive of the intervention being used and may have more readily encouraged participants to engage in mental health services, or led to less honest responses from the participants. Interviews were conducted after a client refused or accepted the intervention, however, and no clients reversed their position during the course of a follow-up interview or immediately following one. Many of the men who accepted mental health treatment were receiving services for other medical problems at the time or had exposure to other mental health programs. All eight men interviewed at the *yeikthar* accepted CETA, and the other seven men were receiving care or services from local clinics or CBO’s. As such, the group of men who accepted are functionally a clinical sample, which presents another potential source of bias. While there were also decliners receiving medical care for other conditions, additional research could clarify the role of willingness to participate in health care programs, and how it interacts with masculinity in novel cultural contexts. Lastly, political prisoners represent a unique group with unique experiences especially as they relate to exposure to potentially traumatic events and subsequent mental health needs. The generalizability of the findings to other traumatized populations is subsequently limited.

## Conclusions

This article explores the relationship between masculinity and mental health treatment seeking among former political prisoners in Yangon, Myanmar. While masculinity was described in consistent terms between groups, there is a notable difference in interpretation of masculine norms when comparing men who accepted and men who declined therapy. This qualitative investigation highlights the need for deeper understanding of what might influence that decision-making process, and how men’s conceptualization of masculine characteristics can help or hinder the likelihood of treatment seeking among traumatized groups. Future research may benefit from examining and possibly reconceptualizing how men are approached, recruited for, and informed about MHPSS programs. Incorporating men’s perspectives on values and beliefs with mental health treatment materials may improve the likelihood of both treatment engagement and retention in mental health treatment. Specifically, research that incorporates men’s own perspectives on positive masculine values with descriptions of mental health programming and evaluation strategies warrants further consideration.

## Data Availability

The original transcripts and interpretation frameworks generated and/or analyzed during this investigation are not publicly available due to the presence of potentially identifiable information. Anonymized transcripts can be provided by the corresponding author by reasonable request.

## Abbreviations

AAPP: Assistance Association for Political Prisoners
CBO: Community-based organization
CETA: Common Elements Treatment Approach
LMIC: Low- and middle-income countries
MHAP: Mental Health Assistance Program
MHPSS: Mental Health and Psychosocial Services
NGO: Non-governmental organization
PTS: Posttraumatic stress
TOT: Trainer-of-trainers
